# c-Ski Inhibits Autophagy of Vascular Smooth Muscle Cells Induced by oxLDL and PDGF

**DOI:** 10.1371/journal.pone.0098902

**Published:** 2014-06-02

**Authors:** Jun Li, Li Zhao, Ting Yang, Yi-Jun Zeng, Kang Yang

**Affiliations:** 1 Department of Cardiothoracic Surgery, Southwest Hospital, Third Military Medical University, Chongqing, China; 2 Department of Biochemistry and Molecular Biology, Third Military Medical University, Chongqing, China; University of Sassari, Italy

## Abstract

Autophagy is increasingly being recognized as a critical determinant of vascular smooth muscle cell (VSMC) biology. Previously, we have demonstrated that c-Ski inhibits VSMC proliferation stimulated by transforming growth factor β (TGF-β), but it is not clear whether c-Ski has the similar protective role against other vascular injury factors and whether regulation of autophagy is involved in its protective effects on VSMC. Accordingly, in this study, rat aortic A10 VSMCs were treated with 40 µg/ml oxidized low-density lipoprotein (oxLDL) or 20 ng/ml platelet-derived growth factor (PDGF), both of which were autophagy inducers and closely related to the abnormal proliferation of VSMCs. Overexpression of c-Ski in A10 cells significantly suppressed the oxLDL- and PDGF- induced autophagy. This action of c-Ski resulted in inhibiting the cell proliferation, the decrease of contractile phenotype marker α-SMA expression while the increase of synthetic phenotype marker osteopontin expression stimulated by oxLDL or PDGF. Inversely, knockdown of c-Ski by RNAi enhanced the stimulatory effects of oxLDL or PDGF on A10 cell growth and phenotype transition. And further investigation found that inhibition of AKT phosphorylation to downregulate proliferating cell nuclear antigen (PCNA) expression, was involved in the regulation of autophagy and associated functions by c-Ski in the oxLDL- and PDGF-stimulated VSMCs. Collectively, c-Ski may play an important role in inhibiting autophagy to protect VSMCs against some harsh stress including oxLDL and PDGF.

## Introduction

Vascular smooth muscle cells (VSMCs) are primary constituents of the blood vessel wall and essential regulators of vascular function. In physiological condition, VSMCs help to maintain vascular tone, regulate blood flow and the oxygen and nutrients distribution. However, during atherogenesis and arterial restenosis, the biology of VSMCs is disordered. VSMCs change from a contractile phenotype to a synthetic phenotype, proliferate abnormally, synthesize extracellular matrix proteins and migrate to the intima, which play a key role in the intimal hyperplasia and progression of the vascular injury [1], [2].

Autophagy is an evolutionarily conserved process involved in the degradation of unnecessary or dysfunctional cellular components, which is a highly inducible and finely controlled cellular event [3], [4]. During autophagy, the cytosolic form of microtubule-associated protein 1 light chain 3–1 (LC3-I) is converted to the phosphatidylethanolamine-conjugated form of LC3 (LC3-II) to promote the formation of autophagosome. Accordingly, the increase of expression and turnover of LC3-II has been widely used to indicate activation of autophagy [5], [6]. Although in early 1960s, the evidence of autophagy occurring in VSMCs in cardiovascular disease was reported [7], [8], only now are the important effects of autophagy on regulating VSMC function in the development of vascular diseases appreciated. Multiple vascular injury risk factors, such as reactive species, cytokines and growth factors can induce autophagy in VSMC, which closely regulate the phenotype, proliferation, plasticity and survival of VSMC to affect the formation of intimal hyperplasia and the composition and stability of vascular lesions [9]–[12].

c-Ski, a homologue of v-Ski in cells, is a versatile transcriptional regulator that is widely distributed in different cells, such as skeleton muscle cells, tumor cells, fibroblasts and VSMCs [13]–[17]. Previously, we have found it can suppress VSMC proliferation stimulated by transforming growth factor β (TGF-β) [18]. But it is not known whether the effect of c-Ski is universal for other risk factors of atherogenesis and arterial restenosis and whether regulation of autophagy is involved in the c-Ski-mediated protective effect in VSMCs. Accordingly, in the present study, we investigated the role of c-Ski in the autophagy and associated VSMC proliferation in rat aortic A10 cells treated with two primary atherogenesis and arterial restenosis regulators and identified VSMC autophagy inducible factors, oxidized low-density lipoprotein (oxLDL) or platelet-derived growth factor (PDGF) [19], [20].

## Materials and Methods

### c-Ski overexpression and RNAi

To overexpress or knock down c-Ski, an adenovirus encoding c-Ski (named Adc-Ski) or target-specific siRNAs (small interfering RNAs) against c-Ski (named c-Ski AdRNAi) were constructed as described previously [18]. AdNull or Scramble RNAi sequence was served as a negative control for Adc-Ski or c-Ski AdRNAi respectively.

### Cell culture and treatment

Rat aortic A10 VSMCs were obtained from the American Tissue Culture Collection and grown as recommended in DMEM modified to contain 4 mmol/L L-glutamine, 4.5 g/L glucose, 1 mmol/L sodium pyruvate, and 1.5 g/L sodium bicarbonate supplemented with 10% fetal bovine serum (FBS) and antibiotics.

To induce autophagy, A10 cells were serum-starved for 24 h and then treated with 40 ug/ml oxLDL (Yiyuanbiotechnology, China) or 20 ng/ml PDGF (R&D Biosystems) for 24 h according to the references [19], [20]. In order to detect c-Ski effect on the oxLDL- or PDGF-induced autophagy, A10 cells were infected with either Adc-Ski or c-Ski AdsiRNA (3×10^4^ particles/cell in 2% FBS for 12 h before starvation), which has been confirmed to overexpress or knockdown c-Ski in A10 cells efficiently [18]. In addition, autophagy inhibitor 3-methyladenine (3-MA)(Sigma) was used to be a positive control for autophagy inhibition in these experiments and AKT inhibitor AKTI IV (Sigma) was used to investigate the associated signaling of c-Ski.

### Cell proliferation assay

The proliferation of A10 cells was determined by Cell Count Kit-8 (CCK-8 Kit, Beyotime Inst Biotech, China) using WST-8(2-(2-methoxy-4-nitrophenyl)-3- (4-nitrophenyl)-5-(2,4-disulfophenyl)-2H-tetrazolium, monosodium salt) dye method as described previously [16], [18].

### Western blotting

To investigate the regulation of autophagy, phenotype transition and associated signaling by c-Ski overexpression or silence in oxLDL- or PDGF-treated A10 cells, western blot analysis with standard procedure was also performed to determine protein expression using antibodies against LC3 (Cell Signaling Technology), α-SMA (Sigma), osteopontin (Santa Cruz), Smad3 (Santa Cruz), phosphor-Smad3 (Thermo Scientific Pierce Products), p-p38 (Santa Cruz), AKT (Cell Signaling Technology), pAKT (Cell Signaling Technology), PCNA(Santa Cruz), p21(Santa Cruz) and p27(Santa Cruz). In these experiments, β-actin (Santa Cruz) served as the endogenous control.

### Statistical analysis

The values are expressed as the means ± SEM. An unpaired Student' *t*-test was used to evaluate the statistical differences between the control and treated groups. In cases of multiple groups, differences were analyzed with one-way ANOVA. Values of *P*<0.05 were considered to be significant. All experiments were repeated at least three times.

## Results

### c-Ski inhibits oxLDL and PDGF-induced autophagy in A10 cells

**Figure 1:**
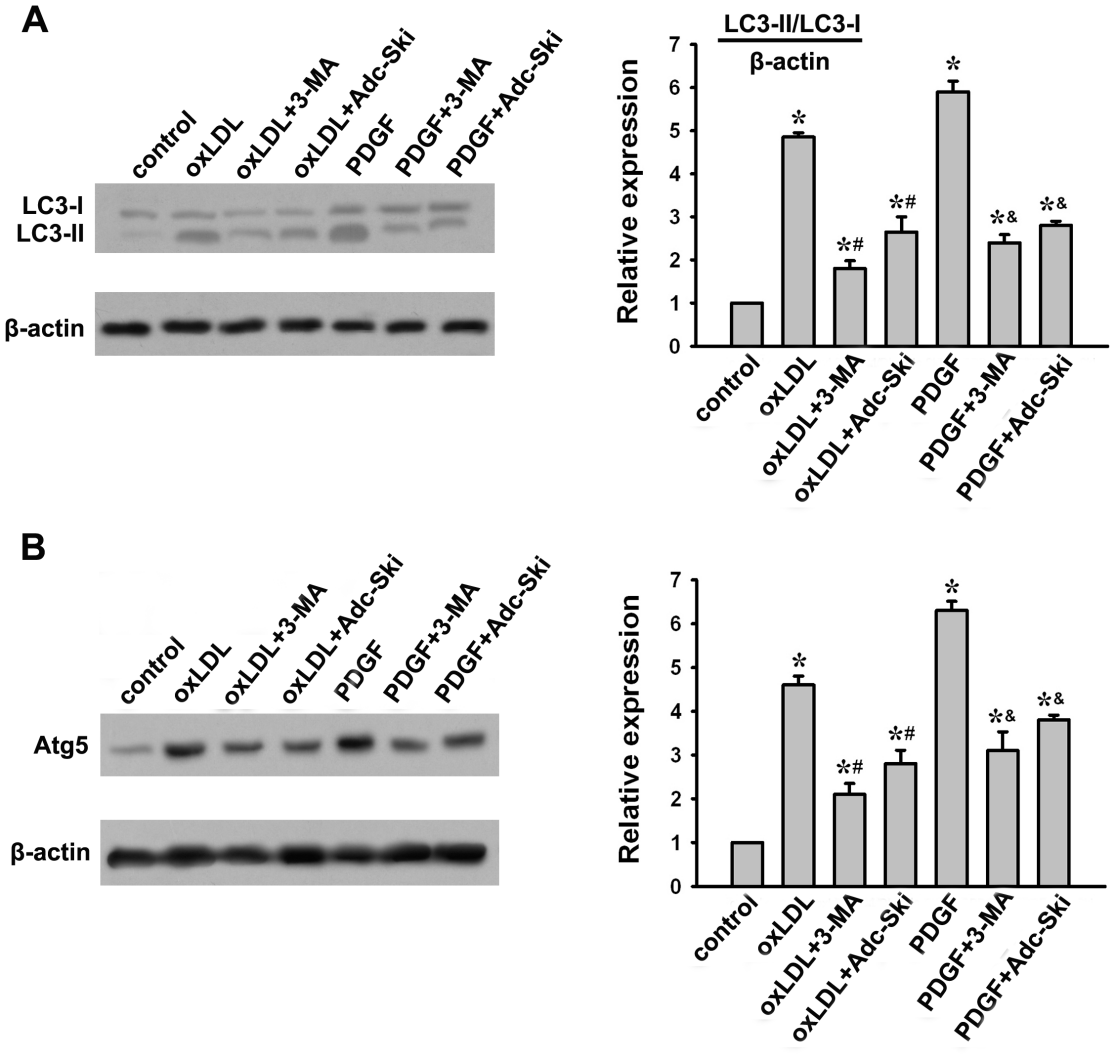
Effect of c-Ski on oxLDL- or PDGF-induced autophagy in A10 cells. c-Ski in rat A10 cells was overexpressed by Adc-Ski transfection. A10 cells were infected with Adc-Ski followed by stimulation with 40 µg/ml oxLDL or 20 ng/ml PDGF for 24 h. As a marker of autophagy, the abundance of LC3 was detected by Western blotting. (A) Effect of c-Ski on oxLDL- or PDGF-induced LC3 expression. (B) Effect of c-Ski on oxLDL- or PDGF-induced Atg5 expression. β-actin served as a control. Bar graphs represent data in mean±SEM based on 3 experiments, *: *P*<0.01 when compared with the control group; #: *P*<0.01 when compared with the oxLDL-treated group; &: *P*<0.01 when compared with the PDGF-treated group.

Both oxLDL and PDGF have been reported to have the ability to induce autophagy in VSMCs [19], [20]. The conversion of LC3-I into LC3-II is an essential step in autophagosome formation, and the abundance of LC3-II correlates with the number of autophagosomes [21]. In addition, Atg5 is an essential autophagy associated gene, which is also used as a marker for autophagy. Accordingly, we assessed the effect of c-Ski on the ox-LDL or PDGF-induced autophagy in A10 cells by measuring LC3-I/LC3-II and Atg5 expressions. As shown in Fig. 1, both ox-LDL and PDGF treatment increased LC3-II and Atg5 expressions significantly. However, these induction of LC3-II and Atg5 were markedly reduced by c-Ski, which to some extent mimicked the effects of autophagy inhibitor 3-MA in response to oxLDL- and PDGF-induced autophagy in VSMCs.

### Inhibition of autophagy by c-Ski affects the proliferation of A10 cells stimulated by oxLDL and PDGF

**Figure 2:**
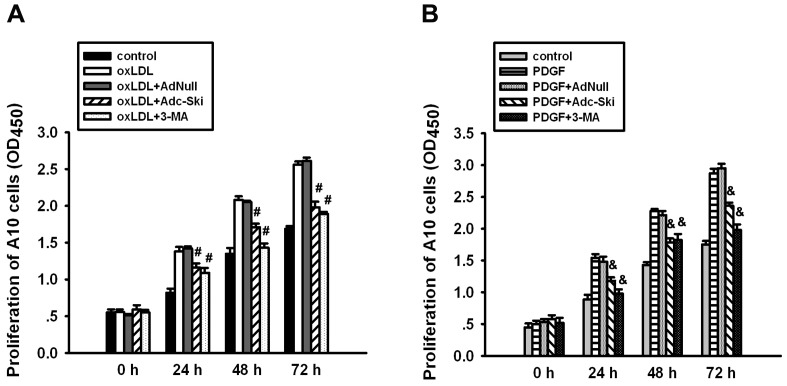
Effect of c-Ski on oxLDL- or PDGF-induced proliferation of A10 cells. The proliferation of A10 cells was assayed using a CCK-8 Kit with WST-8 dye method at 0 h, 24 h, 48 h and 72 h after oxLDL or PDGF treatment. AdNull was served as a negative control for Adc-Ski transfection and 3-MA was used to confirm the autophagy was involved in the proliferation of A10 cells stimulated by oxLDL or PDGF. The representative results are from 3 separate experiments, and the data are expressed as the mean±SEM. #: *P*<0.01 when compared with the oxLDL-treated group at the same time point; &: *P*<0.01 when compared with the PDGF-treated group at the same time point.

Autophagy in VSMCs usually affects the biology of VSMCs, such as proliferation, plasticity, phenotype and survival. As shown in Fig. 2, oxLDL and PDGF stimulated the proliferation of A10 cells, which could be inhibited by 3-MA. It indicates that oxLDL- or PDGF-induced autophagy causes VSMC proliferation. Similar to 3-MA, overexpression of c-Ski by Adenovirus significantly suppressed the proliferation of A10 cells induced by oxLDL or PDGF, which suggested that c-Ski has the potential to inhibit the oxLDL- or PDGF-induced proliferation in VSMCs by suppressing the autophagy.

### Inhibition of autophagy by c-Ski suppresses the PDGF-induced phenotype transition of A10 cells

**Figure 3:**
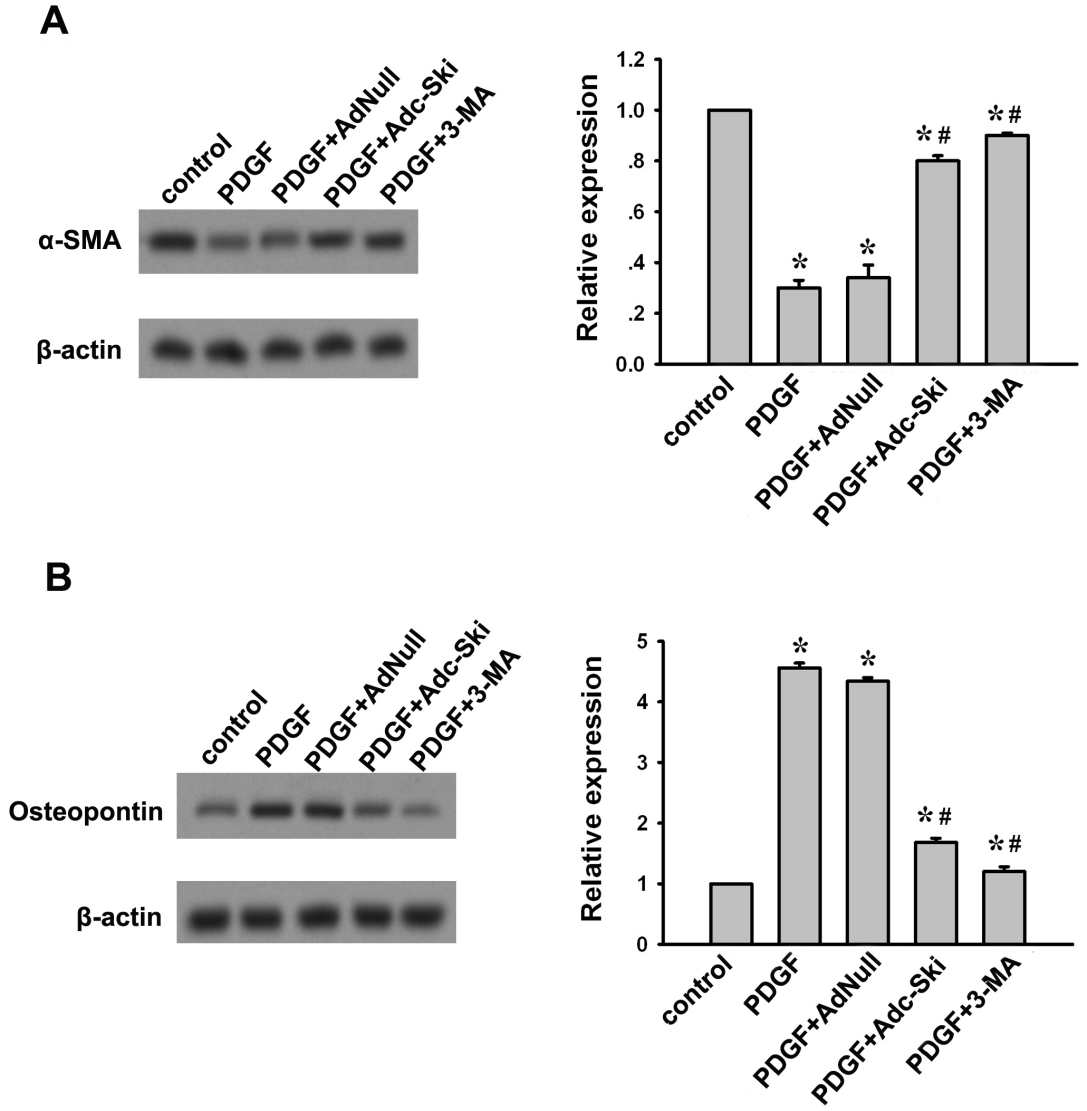
Effect of c-Ski on PDGF-induced phenotype transition of A10 cells. α-SMA is a marker of the contractile phenotype while osteopontin is associated with a synthetic phenotype. Western blot was used to analyze the effect of c-Ski on the contractile and synthetic proteins after PDGF stimulation. (A) Western blot for α-SMA expression. (B) Western blot for Osteopontin expression. Bar graphs represent data in mean±SEM based on 3 experiments, *: *P*<0.01 when compared with the control group; #: *P*<0.01 when compared with the PDGF-treated group.

When VSMCs are exposed to vascular injury factors, they are usually subject to phenotype modulation, that is, phenotype modification from the contractile to the synthetic phenotype as a response to microenvironmental changes. We found in A10 cells, PDGF treatment for 24 h resulted in a decrease in the protein level of α-SMA (Fig. 3A) and a up-regulation of osteopontin (Fig. 3B), which could be blocked by 3-MA. This result confirms previous findings that PDGF promotes the phenotypic transition of VSMCs via autophagy [20]. However, oxLDL was not observed to have obvious effect on α-SMA and Osteopontin expressions in A10 cells (data not shown). Therefore, we further assayed the affects of c-Ski on PDGF-induced VSMC phenotype transition. Similar to 3-MA, pretreatment of Adc-Ski suppressed PDGF-induced decrease of α-SMA and increase of Osteopontin (Fig. 3). It indicates that c-Ski may inhibit VSMCs to change from contractile to synthetic phenotypes which was caused by PDGF-induced autophagy.

### c-Ski RNAi strengthens oxLDL and PDGF-induced autophagy and associated effect on A10 cells

**Figure 4:**
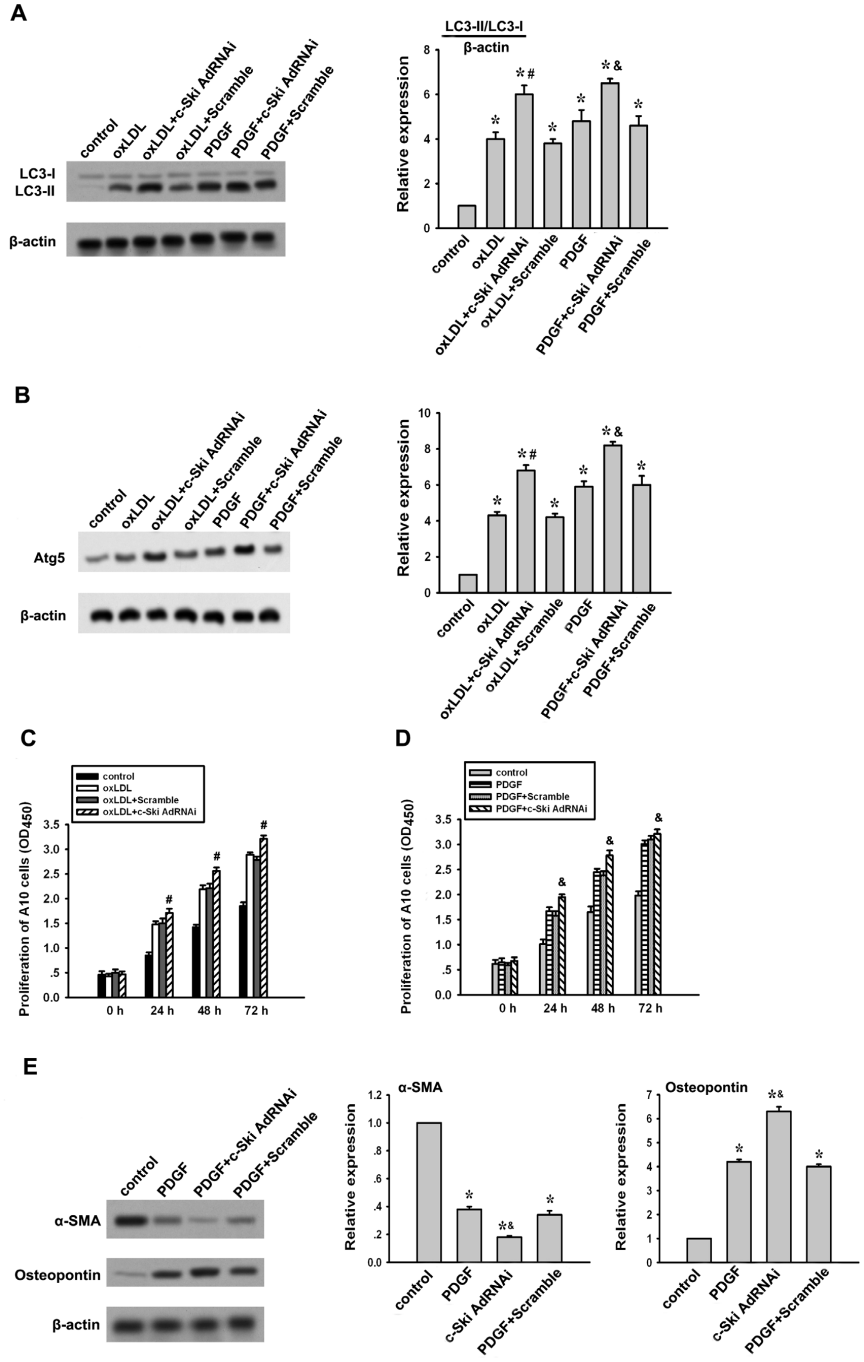
Effect of c-Ski RNAi on oxLDL- or PDGF-induced autophagy and associated functions in A10 cells. c-Ski in rat A10 cells was knocked down by c-Ski AdRNAi transfection, the effects of which on oxLDL- or PDGF-induced autophagy, proliferation and contractile and synthetic protein expressions were detected as described above. Scramble sequence for RNAi was served as a negative control. (A) Effect of c-Ski AdRNAi on oxLDL- or PDGF-induced LC3 expression. (B) Effect of c-Ski AdRNAi on oxLDL- or PDGF-induced Atg5 expression. (C) Effect of c-Ski AdRNAi on A10 cell proliferation stimulated by oxLDL. (D) Effect of c-Ski AdRNAi on A10 cell proliferation stimulated by PDGF. (E) Effect of c-Ski AdRNAi on α-SMA and Osteopontin expressions after PDGF treatment. Bar graphs represent data in mean±SEM based on 3 experiments. *: *P*<0.01 when compared with the control group; #: *P*<0.01 when compared with the oxLDL-treated group; &: *P*<0.01 when compared with the PDGF-treated group.

To confirm the role of c-Ski on oxLDL or PDGF-induced autophagy and associated effect on VSMCs, c-Ski RNAi as previously described [18] was used to knock-down the endogenous c-Ski expression in A10 cells. As shown in Fig. 4A and 4B, c-Ski AdRNAi transfection enhanced oxLDL and PDGF-induced LC3-II and Atg5 expressions. This effect was not observed in A10 cells transfected with scramble sequence. In addition, the proliferation of A10 cells stimulated by oxLDL or PDGF was also strengthened by c-Ski RNAi (Fig. 4C and 4D). Moreover, c-Ski AdRNAi transfection further down-regulated α-SMA expression but up-regulated osteopontin expression in PDGF-treated A10 cells (Fig. 4E). These results confirm that c-Ski has an inhibitory role in oxLDL and PDGF-induced autophagy and associated effect on VSMCs.

### Inhibition of oxLDL and PDGF-induced autophagy and A10 cell proliferation by c-Ski is in a Smad3 and p38-independent manner

**Figure 5:**
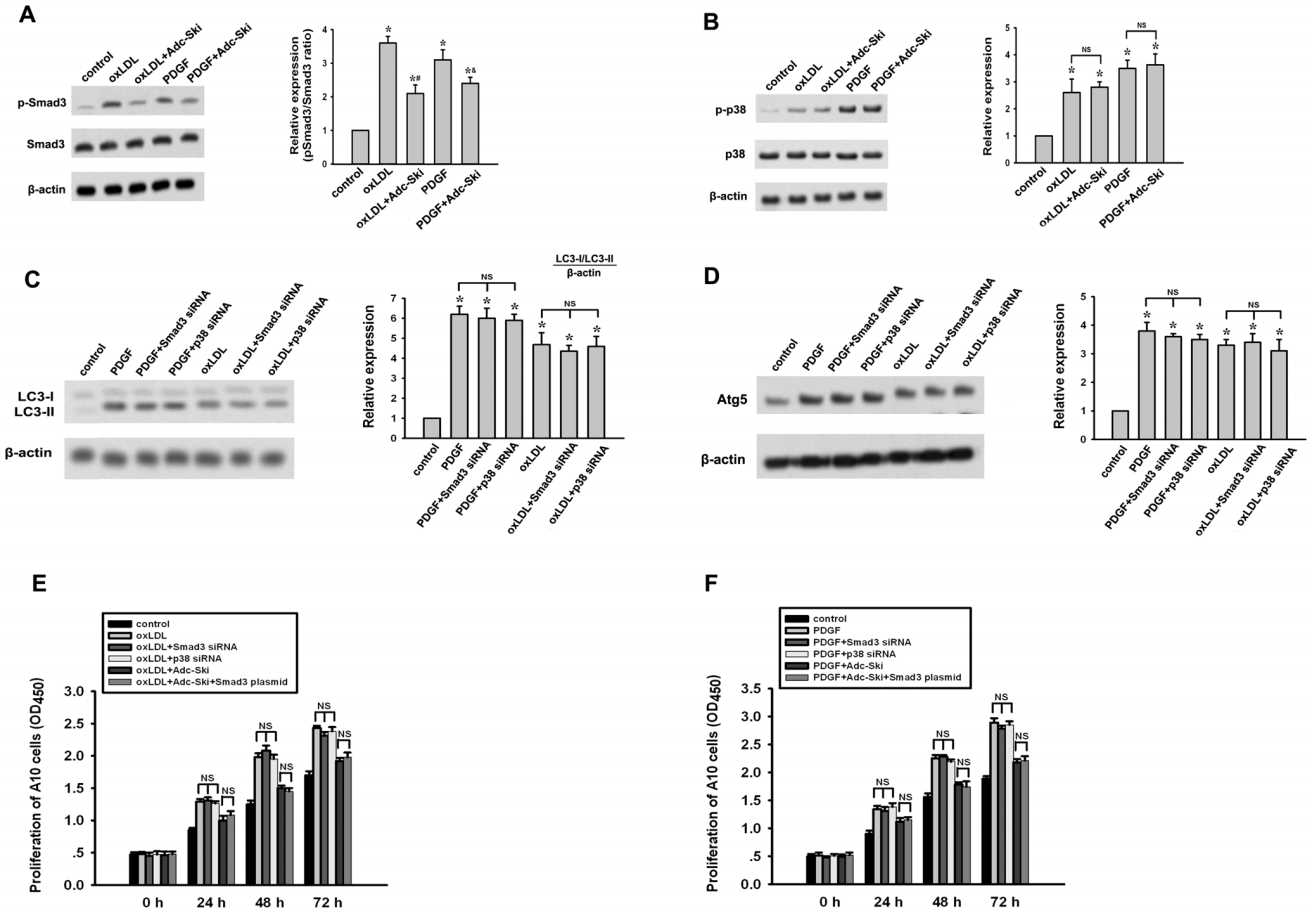
Detection of Smad3 and p38 activation in the inhibitory effect of c-Ski on the autophagy induced by oxLDL or PDGF in A10 cells. Previously, suppression of Smad3 phosphorylation while activation of p38 was demonstrated to be associated with the effect of c-Ski on TGF-β-stimulated A10 cell. Accordingly, to investigate whether this mechanism was involved in the effects of c-Ski on A10 cell treated with oxLDL or PDGF, Smad3 and p38 activation were assayed by western blot. In addition, overexpression of Smad3 by Smad3 plasmid transfection was used to detect whether it could block the effect of c-Ski on oxLDL or PDGF-induced autophagy. (A) Western blot for p-Smad3 and total Smad3. (B) Western blot for p38 and p-p38. (C) Western blot for LC3. (D) Western blot for Atg5. (E) A10 cell proliferation stimulated by PDGF. (F) A10 cell proliferation stimulated by PDGF. Bar graphs represent data in mean±SEM based on 3 experiments. *: *P*<0.01 when compared with the control group; #: *P*<0.01 when compared with the oxLDL-treated group; &: *P*<0.01 when compared with the PDGF-treated group; NS: not significant.

Previously, we have reported that c-Ski can inhibit TGF-β-stimulated VSMC proliferation via suppressing Smad3 phosphorylation but inducing p38 activation [18]. Accordingly, we next investigated whether the Smad3 and p38 signaling were involved in the action of c-Ski in regulating oxLDL or PDGF-induced autophagy. In A10 cells treated with oxLDL or PDGF, the increases of both phosphorylation of Smad3 and action of p38 were detected (Fig. 5A and 5B). However, the Smad3 and p38 activation were not shown to mediate the oxLDL- or PDGF-induced autophagy and associated A10 cell proliferation (Fig. 5C, 5D, 5E and 5F). c-Ski inhibited pSmad3 expression to some extent while exerted no obvious effect on p38 activation (Fig. 5A and 5B). In addition, overexpression of Smad3 by Smad3 plasmid transfection could not block the inhibitory effect of c-Ski on oxLDL- and PDGF-induced LC3-II and Atg5 expressions and the proliferation of A10 cells (Fig. 5C, 5D, 5E and 5F). These results suggest that inhibition of oxLDL- and PDGF-induced autophagy and VSMC proliferation by c-Ski is in a Smad3- and p38-independent manner.

### Suppression of AKT pathway to downregulate PCNA expression is involved in the inhibitory effect of c-Ski on oxLDL and PDGF-induced autophagy and proliferation in A10 cells

**Figure 6:**
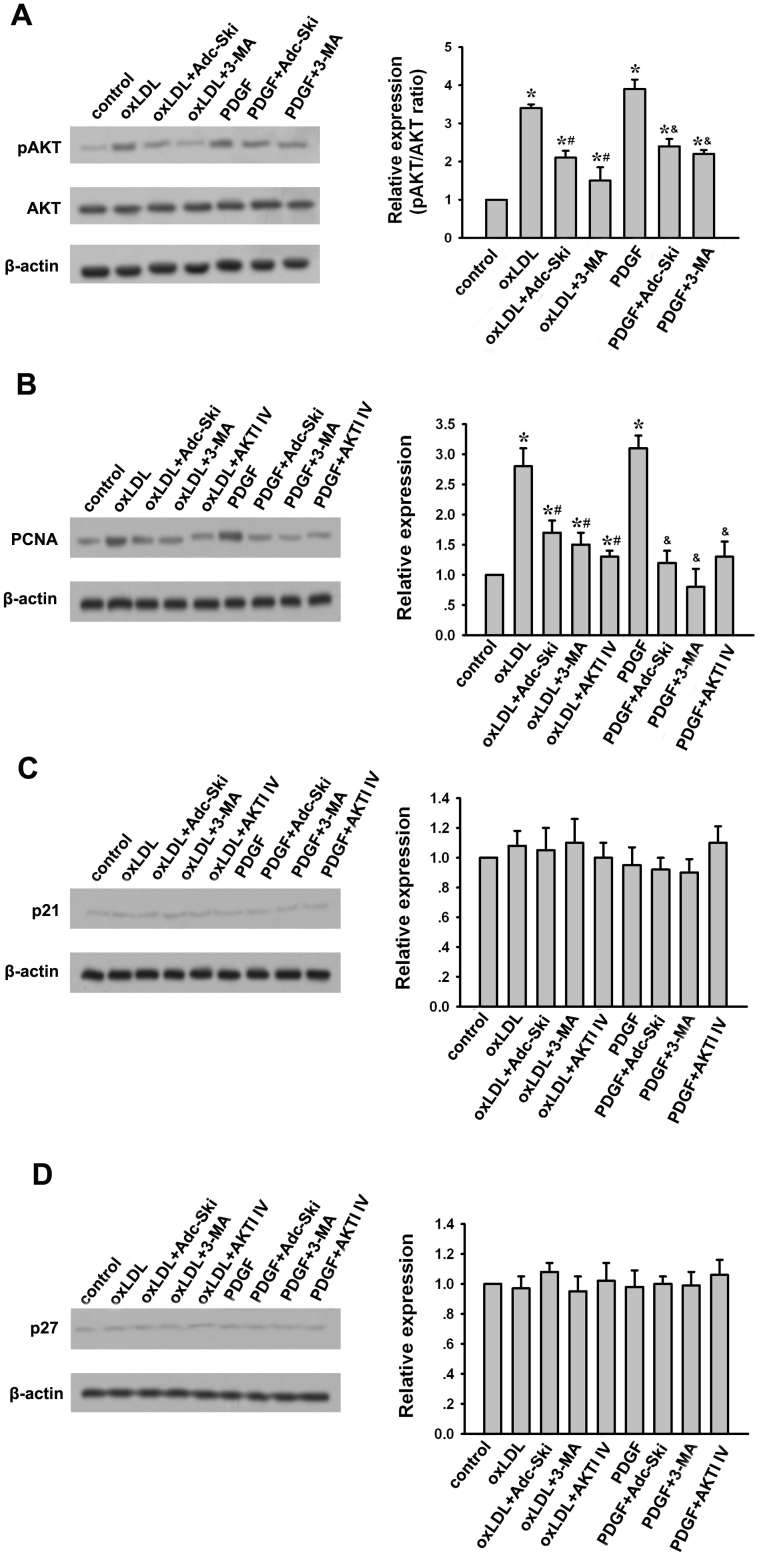
Detection of c-Ski effect on AKT actiation, PCNA, p21 and p27 expressions in the oxLDL- or PDGF-treated A10 cells. (A) Western blot for AKT and pAKT. (B) Western blot for PCNA. (C) Western blot for p21. (D) Western blot for p27. Bar graphs represent data in mean±SEM based on 3 experiments. *: *P*<0.01 when compared with the control group; #: *P*<0.01 when compared with the oxLDL-treated group; &: *P*<0.01 when compared with the PDGF-treated group.

As an autophagy inhibitor, 3-MA inhibits autophagy by blocking autophagosome formation via the inhibition of type III Phosphatidylinositol 3-kinases (PI-3K)-AKT pathway. Since c-Ski exerted similar effect of 3-MA in oxLDL- and PDGF-treated A10 cells as described above, it prompts us to investigate whether AKT signaling is also involved in the action of c-Ski. The results showed that both oxLDL and PDGF significantly increased AKT phosphorylation while like 3-MA, c-Ski could inhibit this inducible AKT phosphorylation efficiently (Fig. 6A). It indicates that suppression of AKT activation may be one mechanism of c-Ski inhibiting oxLDL- and PDGF-induced autophagy. In addition, we found c-Ski could also mimicked 3-MA and AKT inhibitor AKTI IV, suppressing the expression of PCNA stimulated by oxLDL or PDGF but had no effect on p21 and p27 levels (Fig. 6B, 6C and 6D). This may provide an explanation of the inhibitory effect of c-Ski on oxLDL- or PDGF-induced VSMC proliferation.

## Discussion

The present study demonstrates a novel role for c-Ski in regulating autophagy in VSMCs. We found that c-Ski was a robust inhibitor of autophagy induced by oxLDL and PDGF. This inhibitor of autophagy not only significantly suppressed the abnormal proliferation of VSMCs stimulated by oxLDL and PDGF, but also prevented the phenotype transition of VSMCs from the contractile phenotype to the synthetic phenotype in present of PDGF. These findings uncover that c-Ski acts as an important regulator of VSMC proliferation and phenotype by modulating autophagy against the pathological stimulation.

Autophagy, also called “self-eating”, is required to degrade long-lived proteins and dysfunctional organelles under normal conditions, which is a process used for cellular renovation [22], [23]. Realization of the importance of autophagy in VSMCs has only recently emerged. Accumulating evidence suggests that autophagy is activated in VSMCs and it is important for the progression of vascular diseases, such as atherogenesis and arterial restenosis. For example, studies in humans, baboons, monkeys, rabbits, and rats showed convincing Electronic Microscopy (EM) evidence of autophagy in VSMCs occurring in the context of atherosclerosis and hypertension [24]-[30]. In atherosclerosis, basal autophagy is considered a cell survival program for VSMCs, which can attenuate VSMC death under stress and keep the stability of plaques [11], [31]–[34]. However, excessive autophagic activity usually induced by several vascular harsh stimulation, which promotes both loss of the contractile phenotype and increased VSMC proliferation, even leads to total collapse of all cellular functions and induction of autophagic death [20], [35]–[38]. Among these VSMC autophagy stimuli, oxLDL and PDGF are two primary regulators of VSMC growth and proliferation, and play important role in atherogenesis and restenosis [39], [40] . Accordingly, in this study, we used oxLDL and PDGF to induce the autophagy in A10 cells. We found both oxLDL and PDGF induced autophagy in rat arota VSMC A10 cells, which is consistent with the previous reports [19], [20]. But for the first time, we demonstrated that c-Ski, an endogenous molecule in VSMCs, when it was overexpressed, could block oxLDL or PDGF-induced autophagy in VSMCs. This is a novel finding for the function of c-Ski in VSMCs.

Autophagy in VSMCs usually affects the survival and plasticity of VSMC. In the present study, c-Ski significantly inhibited the abnormal proliferation of VSMCs stimulated by both oxLDL and PDGF, and the PDGF-induced contractile to synthetic phenotype transition. Some previous study reported that 187 µg/ml oxLDL reduced the expressions of contractile proteins including α-SMA, smooth muscle myosin heavy chain-1 and calponin in VSMC isolated from Wistar rat aortas [41] whereas treating A7r5 VSMCs with 25 µg/ml oxLDL for 4 days or 50 µg/ml for 1 day could increase synthetic protein expressions such as osteopontin [42]. However, in the present study, we found A10 VSMCs with of 40 µg/ml oxLDL treatment for 24 h did not show obvious changes of α-SMA and osteopontin expressions (data not shown). We speculate the different results may be resulted from the different doses and time of oxLDL treatment or some different characteristics in primary cultured VSMC and different cell lines of VSMC, which need further investigation. Nevertheless, in this study, the effects of c-Ski on inhibiting the abnormal proliferation of VSMCs stimulated by both oxLDL and PDGF, and the PDGF-induced contractile to synthetic phenotype transition could be mimicked by autophagy inhibitor 3-MA, which confirms that regulation of autophagy is involved in the protection of c-Ski in VSMC in present of oxLDL or PDGF stimulation. During atherogenesis and arterial restenosis, VSMCs change from a contractile phenotype to a synthetic phenotype. This promotes their migration to the intima, increases their proliferative capacity and promotes the synthesis of extracellular matrix proteins, then accelerates the vascular pathological progression [43]–[45]. Accordingly, the inhibition of autophagy-associated proliferation and phenotype transition by c-Ski has the potential protection against atherosclerosis. In addition, we also found that in physiological condition, although c-Ski overexpression did not affect the basal autophagy and proliferation of VSMCs obviously, knockdown of c-Ski expression could promote the autophagy and proliferation of VSMCs (Supplementary Fig. 1 and 2). This confirms that c-Ski in VSMC is involved in regulating autophagy and proliferation of VSMCs.

Supporting the beneficial role of c-Ski described above, we previously reported that c-Ski could also inhibit TGF-β-induced VSMC proliferation in vitro and suppressed intimal hyperplasia in a rat balloon injury model [18]. Suppression of TGF-β-stimulated Smad3 phorsphorylation while activation of p38 signaling is demonstrated to mediate the inhibitory effect of c-Ski on TGF-β-induced VSMC proliferation. However, in the present study, Smad3/p38 pathway was not involved in the suppressive action of c-Ski in autophagy and associated effects induced by oxLDL or PDGF in VSMCs. Usually, as a transcriptional co-regulator, c-Ski acts in combination with a number of cellular partners and, thus, has been shown to regulate many signaling pathways, including those mediated by TGF-β, nuclear hormone receptors, and Sonic hedgehog (Shh) [15]. Like PDGF-induced autophagy, Shh-mediated autophagy appeared to support proliferation, suggesting that autophagy may be important to the development or maintenance of the hyperproliferative VSMC phenotype [6]. Interestingly, in accordance with the report that Shh induces this kind of autophagy via triggering AKT signaling, we also detected that activation of AKT is involved in the oxLDL- and PDGF-induced autophagy in A10 cells and c-Ski could inhibit AKT activation to suppress the autophagy. Moreover, we further found like 3-MA and AKTI IV, tow inhibitor of AKT signaling, c-Ski significantly downregulated the PCNA expression induced by oxLDL or PDGF. PCNA is considered to be a marker of cell proliferation, therefore, these findings suggest that AKT-PCNA pathway may mediate the autophagy–induced VSMC proliferation while c-Ski can suppress this pathway to inhibit VSMC proliferation. Although the details of the molecular mechanism need further investigation, c-Ski exerts a potential effect to protect VSMC against several harsh stimulation, such as TGF-β, oxLDL and PDGF. In this view, c-Ski in VSMCs, may serve as a novel target to inhibit or relieve atherosclerosis.

## Conclusion

In summary, the present study demonstrates three novel points: (1) c-Ski inhibits the oxLDL- or PDGF-induced autophagy in VSMCs; (2) c-Ski suppresses the oxLDL- or PDGF-induced proliferation and loss of contractile phenotype of VSMC; (3) These effects of c-Ski on autophagy and associated VSMC biology are in a Smad3/p38-independent manner but associated with AKT-PCNA pathway.

## Supporting Information

Figure S1 **Effect of Adc-Ski and c-Ski AdRNAi on autophagy in A10 cells without stimulation.** A. Western blot for LC3 expression. B. Western blot for Atg5 expression. Bar graphs represent data in mean±SEM based on 3 experiments. *: *P*<0.01 when compared with the control group.
(TIF)

Figure S2 **Effect of Adc-Ski and c-Ski AdRNAi on proliferation of A10 cells without stimulation.** Bar graphs represent data in mean±SEM based on 3 experiments. *: *P*<0.01 when compared with the control group.
(TIF)
