# Supplementary figures and images for: c-Ski Inhibits Autophagy of Vascular Smooth Muscle Cells Induced by oxLDL and PDGF
Source: PLoS One. 2014 Jun 2;9(6):e98902. doi: 10.1371/journal.pone.0098902 (PMC4041777; doi:10.1371/journal.pone.0098902)

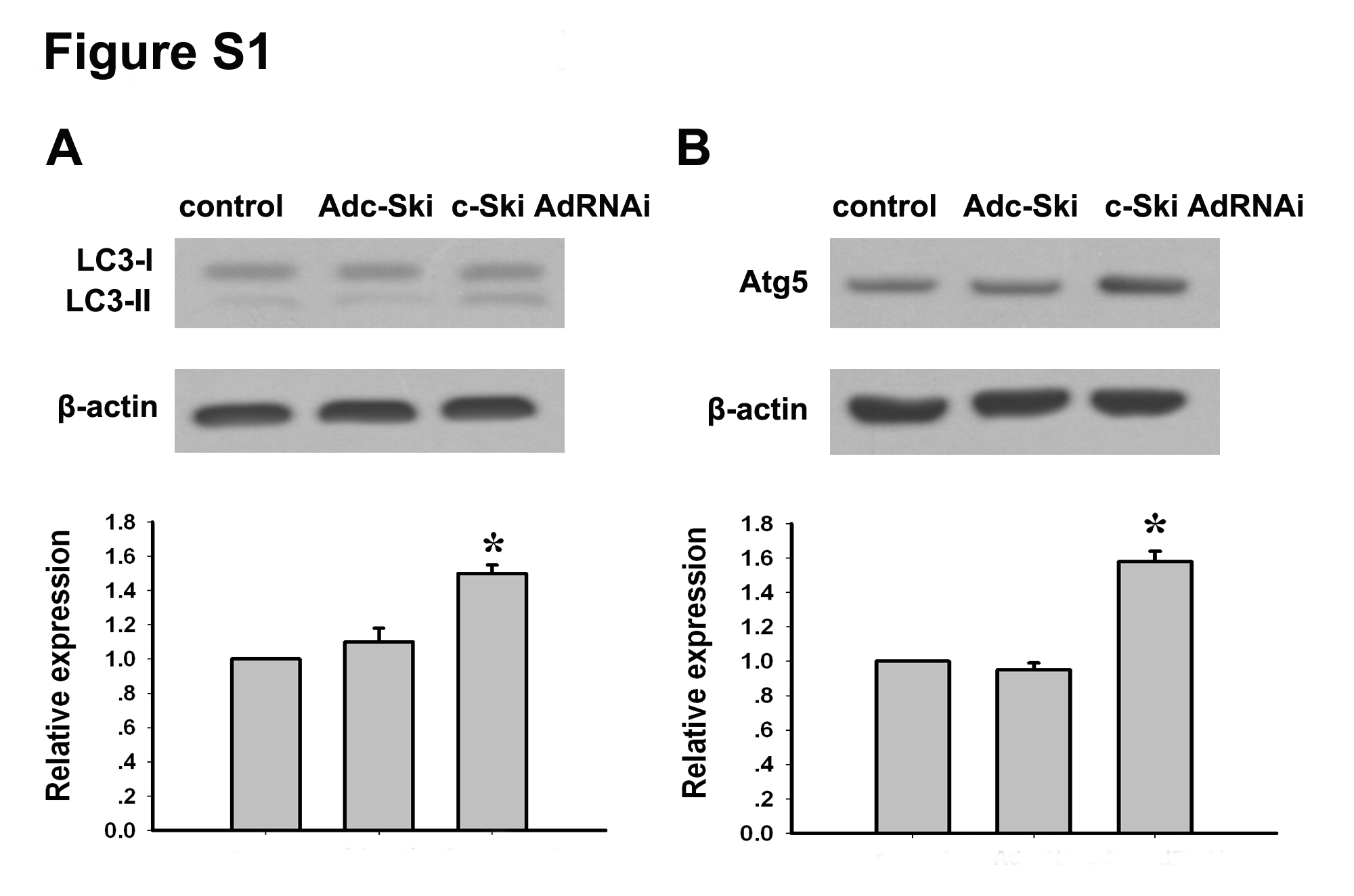

Supplement: Figure S1 — Effect of Adc-Ski and c-Ski AdRNAi on autophagy in A10 cells without stimulation. A. Western blot for LC3 expression. B. Western blot for Atg5 expression. Bar graphs represent data in mean±SEM based on 3 experiments. *: P<0.01 when compared with the control group. (TIF) [file pone.0098902.s001.tif]

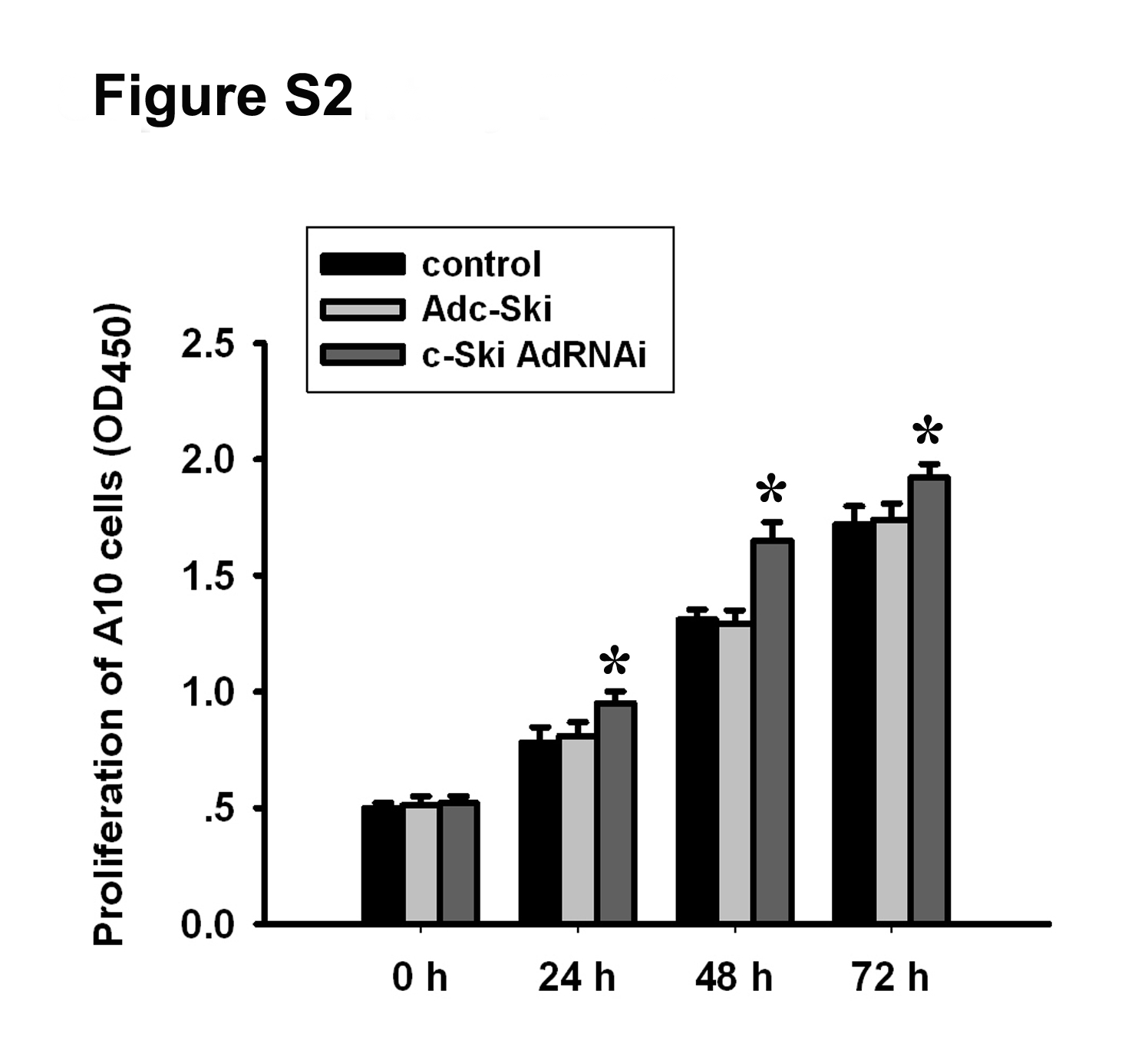

Supplement: Figure S2 — Effect of Adc-Ski and c-Ski AdRNAi on proliferation of A10 cells without stimulation. Bar graphs represent data in mean±SEM based on 3 experiments. *: P<0.01 when compared with the control group. (TIF) [file pone.0098902.s002.tif]
